# Abnormal Posture Relating to the Alignment of Spine and Lower Extremity

**DOI:** 10.1155/2019/8460364

**Published:** 2019-07-25

**Authors:** Yasushi Oshima, Nobuyoshi Watanabe, Shinro Takai, Mitsuhiro Kawata

**Affiliations:** ^1^Department of Orthopaedic Surgery, Nippon Medical School, 1-1-5 Sendagi, Bunkyo-ku, Tokyo 113-8603, Japan; ^2^Department of Orthopaedic Surgery, Kyoto Kujo Hospital, 10 Karahashi Rajomon-cho, Minami-ku, Kyoto 601-8453, Japan; ^3^School of Health Sciences, Bukkyo University, 7 Higashitoganoo-cho, Nakagyo-ku, Kyoto 604-8418, Japan

In an aging society with an increase in the average life expectancy, abnormal posture with body balance disorder is a serious issue because it results in the reduction of activities of daily living and health-related quality of life.

Spinal deformity is an essential factor for adult abnormal posture defined as an abnormality in alignment, formation, or curvature of 1 or more portions of the spine [1]. Adult spinal deformity (ASD) reportedly occurs in more than 60% of the older population and develops with multiple conditions, including trunk muscle weakness, disk degeneration, vertebral fracture, and spondylotic changes. ASD is classified into coronal curve types and three sagittal modifiers (the difference between the pelvic incidence and the lumbar lordosis, the global alignment, and the pelvic tilt) [2]. In ASD, sagittal plane deformity is known to be of critical importance for pain and disability; therefore, recently, posterior spinal fusion has been applied for the correction of sagittal spinal malalignment. However, the surgical indication and the target of the alignment for each patient remain controversial. Besides the varying of the thoracic kyphosis and the lumbar lordosis, the spine anatomically connects to the pelvis involving the hip joint; therefore, ASD management is crucial focus for spino-pelvic-hip alignment.

The hip-spine syndrome was first described by C. M. Offierski, wherein the degenerative change of the hip and the lumbar spine are interrelated [3]. It is classified into 4 types: simple, complex, secondary, and misdiagnosed. Thereafter, the deformity of the spine or the hip and the following compensation mechanisms of each other have been investigated. Consequently, spinal surgery is able to reduce low back pain and abnormal posture directly; however, total hip replacement may also have the potential to repair hip disorders and improve posture. Moreover, the pelvis is known as the main regulator of a chain of correlation between the spine and the lower extremities. Thus, the relationship among the spine, the pelvis, and the hip joint for the treatment of the hip-spine syndrome needs further investigations.

Similarly, the knee-spine syndrome demonstrates that the symptoms from the lumbar spine may be caused by osteoarthritis (OA) of the knee and that the limitation of knee extension in turn affects the loss of lumbar lordosis [4]. In the treatment of knee pain, the limitation of the knee range of motion, and the malalignment of the lower extremity in the knee OA, many surgical procedures have been developed, including around knee osteotomies, unicompartmental knee arthroplasty, and total knee arthroplasty. All the surgical techniques have shown promising results with respect to the correction of the malalignment of the lower extremities and clinical outcomes. However, the target of the alignment in the lower extremities is different in each surgical procedure. Moreover, the effect on the corrected alignment following each procedure on the posture in older people remains debatable. At least, knee OA is known to affect spinal alignment; and therefore knee treatments can improve posture.

The feet act as a fulcrum, connecting to the lower extremities, pelvis, and spine, and the head moves like a pendulum. Hence, body balance and posture resemble an inverted pendulum, and the degeneration of these parts could affect the posture. Therefore, all these portions should be ultimately considered for the treatment of abnormal posture together although it is an extremely confusing issue.

With this background, in this special issue, we would like to focus on the principle of abnormal posture as well as the management of the malalignment and optimal alignment of the spine and the lower extremities.
